# A Place-based Assessment of Flash Flood Hazard and Vulnerability in the Contiguous United States

**DOI:** 10.1038/s41598-019-57349-z

**Published:** 2020-01-16

**Authors:** Sepideh Khajehei, Ali Ahmadalipour, Wanyun Shao, Hamid Moradkhani

**Affiliations:** 1 Center for Complex Hydrosystems Research, Department of Civil, Construction and Environmental Engineering, University of Alabama, Tuscaloosa, AL USA; 2 Center for Complex Hydrosystems Research, Department of Geography, University of Alabama, Tuscaloosa, AL USA

**Keywords:** Hydrology, Natural hazards

## Abstract

Flash flood is among the most catastrophic natural hazards which causes disruption in the environment and societies. Flash flood is mainly initiated by intense rainfall, and due to its rapid onset (within six hours of rainfall), taking action for effective response is challenging. Building resilience to flash floods require understanding of the socio-economic characteristics of the societies and their vulnerability to these extreme events. This study provides a comprehensive assessment of socio-economic vulnerability to flash floods and investigates the main characteristics of flash flood hazard, i.e. frequency, duration, severity, and magnitude. A socio-economic vulnerability index is developed at the county level across the Contiguous United States (CONUS). For this purpose, an ensemble of social and economic variables from the US Census and the Bureau of Economic Analysis were analyzed. Then, the coincidence of socio-economic vulnerability and flash flood hazard were investigated to identify the critical and non-critical regions. Results show that the southwest U.S. experienced severe flash flooding with high magnitude, whereas the Northern Great Plains experience lower severity and frequency. Critical counties (high-vulnerable-hotspot) are mostly located in the southern and southwestern parts of the U.S. The majority of counties in the Northern Great Plains indicate a non-critical status.

## Introduction

Flash floods impose extensive damage and disruption to societies, and they are among the deadliest natural hazards worldwide. Several studies have assessed the impacts of flash flood events around the world with regards to substantial financial losses, destruction of infrastructures, displacement, and fatalities^1–3^. Climate change is expected to increase extreme rainfalls and heavy river discharge, which will in turn exacerbate the likelihood of frequent flash floods with amplified severity^4–6^. There is an extensive body of research on the effects of development and construction in riverine floodplains, whereas less attention has been given to flash flooding and its impacts^7,8^.

The compound effects of population growth, inappropriate land-use planning and management, and environmental degradation altogether with the impacts of climate change are the main drivers of increasing losses triggered by the incident of natural hazards^9–11^. Flash flood is identified as a natural hazard with the highest capacity to generate damages to the human society on a global scale^12,13^. The high risk associated with the flash flood is due to its main characteristic: rapid onset and happening in a relatively short time, which significantly limits the warning and response time of the affected population and concerned agencies^14,15^.

Compound effect of the sensitivity of a population and its capability to respond to and recover from the consequences of a natural hazard construct the socio economic vulnerability^16,17^. Socioeconomic vulnerability is an integral variable that is influenced by a variety of factors, and thus it is essential to understand the societies’ characteristics and investigate their susceptibility to the hazards’ impact^18–20^.

Despite the significant advances in defining and formulating vulnerability^21–24^, there still remains disparities in interpretation of vulnerability between the risk/hazard research and human-environmental research communities. However, both communities acknowledge that vulnerability is composed of exposure, sensitivity, and response or resilience to the compound effects of both human and environmental systems^25–28^.

Vulnerability analyses are mainly classified into two groups that either (1) investigate the natural hazard characteristics and the physical attributes that aggravate the loses, which can be summarized as biophysical vulnerability, or (2) address the compound effect of socio-economic status and exposure, which directly affect the impacts^29^. Multitude of studies have focused on the elements of exposure due to less complexity compared to the socio-economic vulnerability. However, socio-economic vulnerability can address the root causes of damages to societies by investigating the social status, economic growth, population limitations, and several other characteristics^16^. Several other authors have discussed, in much greater details, many of the important aspects of social vulnerability and its measures^23,27,30,31^.

A region with advanced socio-economic status, and thus more efficient coping mechanisms in place, is generally less vulnerable to disasters^32^. Indicators of social and economic status often include percentage of vulnerable population such as the youth and the elderly, per capita income, extent of access to public amenities, and other socio-economic variables^33,34^. To this end, an index-based vulnerability assessment is a practical tool that helps comparing and ranking areas in terms of their vulnerability^16,35,36^.

Špitalar *et al*.^37^ evaluated the fatalities from 2006 to 2012 in the United States caused by flash floods and realized most of the fatalities accrued in rural areas. However, when a flash flood occurs in an urban area, human impacts (i.e., injuries and fatalities per event) would be higher. Notably, the frequency and number of flood fatalities are likely to increase over the U.S. in the coming decades due to two primary reasons^15^. Urbanization has increasing trend in the U.S.^38^ and urbanized basins are more prone to impacts from intense rainfall with reduced infiltration and the absence of natural defenses such as vegetation. Second, climate change studies have projected an intensifying hydrologic cycle under future emission scenarios, resulting in more intense rainfall events and exacerbated potential for flash floods^39–42^.

Very few studies have particularly evaluated the vulnerability to flash flood events. Milansei *et al*.^43^ investigated the flash flood vulnerability for risk mitigation of large buildings, whereas Karahiorgos *et al*.^44^ implemented a statistical model to countify vulnerability to flash floods. However, it is essential to investigate the socio-economic status and its interaction. with flash flood characteristics. Such a study can help advance flash flood risk mitigation planning as well as socio-hydrologic management to reduce the adverse impact of flash floods in the United States and beyond. As a result, it is critical to develop a comprehensive indicator of the disaster risk and vulnerability to flash floods. The vulnerability to flash floods depends on both biophysical and socio-economic factors. An indicator of flash flood vulnerability should be able to help the decision makers assess the potential impacts of flash floods and identify the most vulnerable social group and areas^45^.

The current study provides a comprehensive and multi-dimensional assessment of socio-economic vulnerability and its coincidence with flash flood characteristics over the contiguous United States (CONUS). Built upon previous analyses^16^, this study improves the measure of socio-economic vulnerability by utilizing Probabilistic Principal Components Analysis (PPCA) that handles the issue of missing values in the data. We then integrate the socio-economic vulnerability and flash flood characteristics at the county level to analyze the spatial distribution of flash flooding across the CONUS. Therefore, two main components of risk (i.e. vulnerability and hazard) are assessed here for flash floods. The results will indicate the resemblance and heterogeneity of flash flood spatial clustering and vulnerability of the regions over the CONUS. Identifying these spatial patterns will assist policy makers reach informed and effective decisions for planning and allocating resources.

## Data

### Socio-economic data

The categories that develop the basis of Socio-Economic Vulnerability Index (SEVI) in this study are identified as: (1) demographic socioeconomic status, (2) race and ethnicity, (3) age, (4) employment and gender, (5) housing and transportation, and (6) industrial economy. The data from the first five categories are named social variables and are acquired from the 2015 American Community Survey (ACS) 5-year Estimates (https://factfinder.census.gov/faces/nav/jsf/pages/index.xhtml). The industrial economy variables are collected from the Bureau of Economic Analysis (https://www.bea.gov/iTable/index_regional.cfm). Descriptions of the chosen variables are summarized in Table 1.

**Table 1:** Variables used in this study to quantify socio-economic vulnerability index (SEVI).

Categories	Variables	Influence on the Vulnerability
Demographic Socioeconomic Status	Poverty	+
	Per capita income	−
	Median household value	−
	Percentage of population aged 25 years or older with less than 12th grade education	+
	Percentage of households receiving social security	+
	Median gross rent	−
	Percentage employment in extractive industries	+
	Percentage of households earning greater than US $200,000 annually	−
	Percentage employment in service industry	+
	Percentage civilian unemployment	+
Race and Ethnicity	Percentage Asian	+
	Percentage Black or African American	+
	Percentage speaking English as a second language with limited English proficiency	+
	Percentage Hispanic	+
	Percentage Native American	+
Age	Median age	−
	Percentage of population under 5 years or 65 and older	+
	Percentage of population under 18 years old	+
Gender	Percentage female	+
	Percentage female participation in labor force	+
	Percentage female-headed households	+
Housing and Transportation	People per unit	+
	Percentage mobile homes	+
	Percentage of housing units with no cars	+
	Percentage of population living in nursing and skilled-nursing facilities	+
	Percentage renters	+
	Percentage unoccupied housing units	−
Industrial Economy	Private industries	−
	Agriculture, forestry, fishing, and hunting	−
	Transportation and food service	−
	Accommodation and food service	−
	Governmental	−

The last column indicates the overall correlation of each variable to vulnerability (i.e. a positive sign means that increase in variable will increase SEVI, and vice versa).

### Flash flood data

The Unified Flash Flood Database was assembled by the Hydrometeorology and Remote Sensing (HyDROS) group at the University of Oklahoma. The Unified Flash Flood Database is composed of data from a variety of sources including the gauge measurements of streamflow by the United States Geological Survey (USGS), flash flooding reports in the National Weather Service (NWS) Storm Events Database, and public survey responses on flash flood impacts collected during the Severe Hazards Analysis and Verification Experiment (SHAVE)^46,47^.

This study has used the USGS automated streamflow measurements (which is a primary data source in the FLASH database developed by HyDROS) for investigating flash flood hazard characteristics. The flash flood characteristics were extracted from the USGS streamflow measurements collected by the HyDROS for the period of 1950 to 2017 over 3,490 stations. Based on the USGS database, flood events are defined when streamflow exceeds the defined action stage for a particular gauge. These action stages are derived based on the historical streamflow record at the location of interest as well as the physical characteristics of the area and the potential flood protection structures (e.g. levees). The USGS database that is used in this study provides the following information for each gauge: the USGS gauge identifier (ID), latitude (decimal degrees), longitude (decimal degrees), start time (UTC) at which the flow first exceeded the action stage threshold, end time (UTC) when the flow receded below the threshold, peak flow magnitude (cms), peak time (UTC) at which peak flow occurred, and the flood rise time (hr) defined as the difference between the start time and peak time.

This study focuses on four characteristics of flash flood at each station including the frequency, magnitude, duration, and severity. Frequency is extracted from the USGS database based on the number of times that flash flood is reported for a specific station. The peak streamflow is treated as the magnitude, whereas the flood rise time is considered for duration. Severity is defined as the flash flood magnitude divided by the duration.

## Methodology

### Socio-economic vulnerability index (SEVI)

Cutter *et al*.^16^ introduced the Social Vulnerability Index (SoVI) to examine the spatial patterns of social vulnerability to natural hazards at the county level in the United States. The SoVI was developed by collecting the social characteristics consistently identified within the research literature as contributors to vulnerability. These variables should explain the socioeconomic status of the given region as well as its capacity for recovery from the impacts of natural hazards. Selection of the specific variables which represent the socio-economic vulnerability is specific to the study objective. However, the most common characteristics include demographic socioeconomic status, the quality of human settlements, and the built environment^16^. Due to the high number of variables describing the social vulnerability, statistical procedures are commonly used to reduce data dimensions. The Principal Component Analysis (PCA)^48^ has been widely utilized for decreasing the dimension of data in order to create a single and consolidated index of social variables^16,21,49–52^. Based on PCA, the components which explain the majority of the variance in the data will be chosen to calculate the SEVI. The most important step in constructing the index is scaling the chosen components. Each component represents a different element of vulnerability, therefore positive values raise the vulnerability index and negative values reduce it^53^. One shortcoming of using PCA-based index is that this procedure falls short in dealing with missing values. To overcome this problem, a few studies have suggested replacing the missing values with a value of zero^16,21,52^. However, a zero value cannot accurately represent the true vulnerability based on the particular variable of interest, and it would underestimate the level of vulnerability for the affected regions.

This study introduces a new algorithm for building the socio-economic vulnerability, which is based on Probabilistic Principal Components Analysis (PPCA) introduced by Tipping and Bishop^54^, which overcomes the shortcomings of PCA. The PPCA is a probabilistic formulation of PCA based on a Gaussian latent variable model, which has the capability to estimate the missing values, and therefore, there is no need for replacing the missing values with zero. PPCA retains the characteristics of PCA such as the principle scores and loadings. The expectation maximization (EM) algorithm is used to estimate the parameters of PPCA model, which will consequently allow the framework to deal with the missing values^55,56^.

In more detail, PPCA represents a constrained form of Gaussian distribution in which the number of free parameters can be restricted while still allowing the mode to capture the dominant correlation in a dataset. It is expressed as the maximum likelihood solution of a probabilistic latent variable model.

Given a PPCA model M, one can use it to transform observations into latent variables, as:

1 $$z=({W}^{T}W+{\sigma }^{2}\,I){W}^{T}(x-\mu )$$

Where x refers to observation matrix, W is the factor loadings or weight matrix, and I reparenting the identity matrix. The model parameters W, σ, and μ can be estimated by maximizing the likelihood function. For further information on PPCA readers are referred to^54^.

To generate the SEVI, the social and economic variables were chosen based on the previous studies^16,57^, and they are presented in the Data section. These variables include demographic socioeconomic status, race and ethnicity, age, employment and gender, housing and transportation, and industrial economy^20^. The demographic socioeconomic status demonstrates the ability of a society to absorb losses and enhance resilience to hazards^58^. In addition, wealth enables communities to absorb and recover from losses more quickly due to insurance and social safety nets^59^. Race and ethnicity impose language and cultural barriers that may affect access to post-disaster funding and residential locations in hazardous areas^60^. Extreme age spectrum affects mobility. For example, parents spend time and money caring for children when daycare facilities are affected. The potential loss of employment following a disaster exacerbates the number of unemployed workers in a community, contributing to a slower recovery from the disaster. Loss of sewers, bridges, water, communications, and transportation infrastructure compounds potential disaster losses. The loss of infrastructure may place an insurmountable financial burden on smaller communities that lack the financial resources to rebuild^16^. The income from the industries provides an indicator of the state of economic health of the community and longer term issues with recovery after an event^61^.

All the input variables should be normalized at first, therefore the z-scores were calculated for all the data with zero mean and unit standard deviation. The PPCA was performed with the normalized input variables. PPCA returns a set of orthogonal components that are the linear combinations of the original variables. In this step, it is necessary to choose the number of components representing the chosen variables. In this study, the Kaiser criterion^62^ was used to select the number of components, which retains the components with eigenvalues greater than 1. Then, the varimax rotation was applied to the retaining components in order to reduce the number of high-loading variables on each component. Before formulating the SEVI, the resulting components should be scaled based on their influence on the socio-economic vulnerability the influence of the dominating socio-economic factor, as if it has positive or negative sign. The influence of the chosen variables is presented in Table 1. For this step, an output of the loadings of each variable on each factor was used to determine if high levels of a given factor tend to increase or decrease social vulnerability. If a factor tends to show high levels for low social vulnerability (e.g., higher wealth improves the socio-economic status), the corresponding factor scores are multiplied by –1. In some cases, both high and low levels may increase social vulnerability (e.g., a mature population decreases the socio-economic status and abundance of children increases the status) and in this case the absolute value of the corresponding factor score was used for calculating SEVI. Following the suggestion of Schmidtlein *et al*.^53^, the component scores were combined by weighted sum using explainable variance. Finally, the resulting score was normalized between 0 and 1 to calculate the final SEVI.

In summary, the SEVI is calculated using the following steps:

1. Standardize all input variables to z-scores, each with zero mean and unit standard deviation.

2. Perform the PPCA with the standardized input variables.

3. Select the number of components to be further used based on the unrotated solution.

4. Rotate the initial PPCA solution.

5. Interpret the resulting components on how they may influence the social vulnerability and assign signs to the components accordingly.

6. Combine the selected component scores into a univariate score using weighting scheme on the explained variance.

7. Standardize the resulting scores to mean 0 and standard deviation 1.

**Figure 1:**
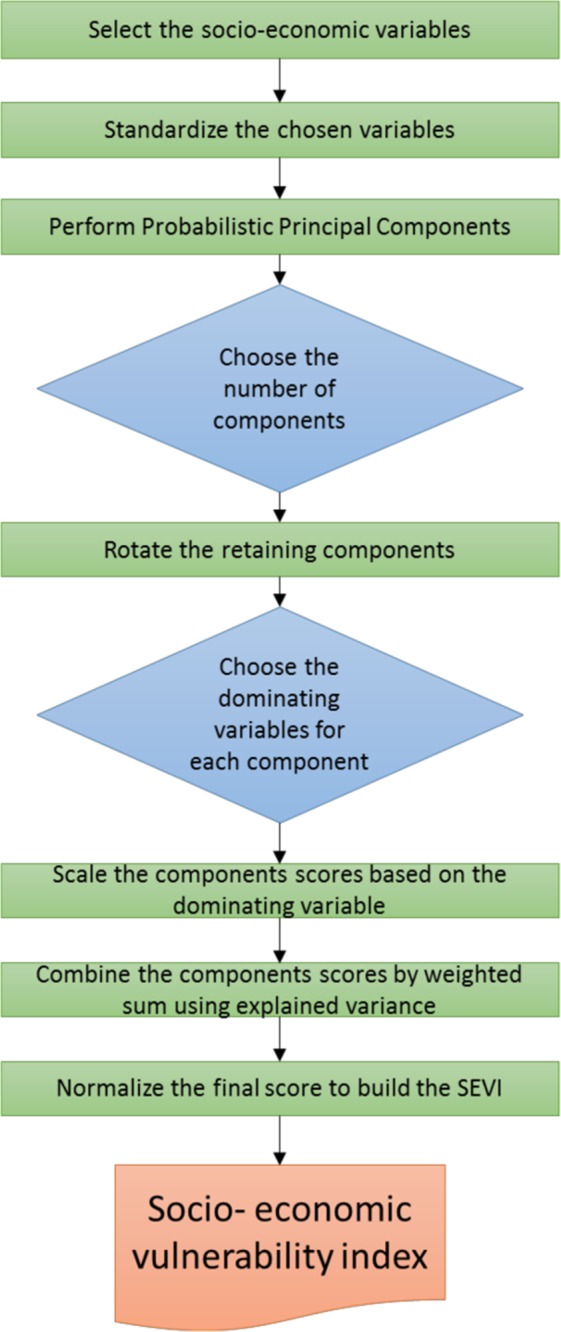
The methodology employed to calculate the Socio-Economic Vulnerability Index (SEVI).

Figure 1 presents the schematic flowchart for SEVI development procedure in this study.

The methodology was applied to the socio-economic variables at county level, and for clear presentation, the regional units (i.e. counties) were divided into three categories; low vulnerable (SEVI <= 10^th^ Percentile), medium vulnerable (10^th^ Percentile < SEVI < 90^th^ Percentile) and high vulnerable (SEVI => 90^th^ Percentile). The calculated socio-vulnerability is a unitless spatial measure, and it is designed to be employed as a comparative value across geographic locations.

### Flash flood clustering

The United States Geological Survey (USGS) stations were spatially clustered based on each flash flood characteristic (i.e., magnitude, duration, frequency, and severity). This study has employed the Getis-Ord hotspot analysis for this purpose^63^. The Getis-Ord hotspot analysis is a parametric technique which can capture the local hotspots. The hotspot analysis uses the flash flood characteristics to identify the locations of statistically significant hot spots and cold spots in the USGS data.

A high Z-score and small P value for a region indicates a significant hot spot (i.e. significant clusters of high values). A low negative Z-score with a high absolute value and small P value indicates a significant cold spot (i.e. significant clusters of low values). In simple words, hotspots are the regions that the variable of interest (e.g. flash flood magnitude or frequency) is high and the surrounding regions also indicate large values. Therefore, they are associated with higher hazard. The higher the Z-score (absolute value), the denser is the clustering. A Z-score near zero means no spatial clustering.

Give the station i, in total number of n stations, the Getis-Ord local statistic is given as:

2 $${({\rm{Z}}-{\rm{score}})}_{i}=\frac{{\sum }_{{\rm{J}}=1}^{{\rm{n}}}{{\rm{w}}}_{{\rm{i}},{\rm{j}}}{{\rm{x}}}_{{\rm{j}}}-\bar{{\rm{X}}}{\sum }_{{\rm{J}}=1}^{{\rm{n}}}{{\rm{w}}}_{{\rm{i}},{\rm{j}}}}{{\rm{S}}\sqrt{\frac{[{\rm{n}}{\sum }_{{\rm{J}}=1}^{{\rm{n}}}{{\rm{w}}}_{{\rm{i}},{\rm{j}}}^{2}-{({\sum }_{{\rm{J}}=1}^{{\rm{n}}}{{\rm{w}}}_{{\rm{i}},{\rm{j}}})}^{2}]}{{\rm{n}}-1}}}$$

Where x_j_ is the attribute value for feature j, w_i,j_ is the spatial weight (i.e, the distance between the stations) between stations i and j, and n is the total number of stations and:

3 $$\bar{{\rm{X}}}=\frac{{\sum }_{{\rm{J}}=1}^{{\rm{n}}}{{\rm{x}}}_{{\rm{j}}}}{{\rm{n}}}$$

4 $${\rm{S}}=\sqrt{\,\frac{{\sum }_{{\rm{J}}=1}^{{\rm{n}}}{{\rm{x}}}_{{\rm{j}}}^{2}}{{\rm{n}}}-{(\bar{{\rm{X}}})}^{2}}$$

## Results and Discussion

The results of socio-economic vulnerability analysis and its spatial relation with flash flood characteristic are divided into two sections. The first section investigates the characteristics of SEVI over the U.S. counties. Then, the resemblance of the flash flood characteristics and the SEVI is evaluated over the CONUS.

### Socio-Economic Vulnerability over the CONUS

The SEVI is calculated by feeding the PPCA with the 32 social and economic variables described in section 2.1. The PPCA analysis of socioeconomic vulnerability resulted in retaining nine components, which explain 78% of the total variance among the U.S. counties. Each of the components explains between 3 to 17 percent of the total variance. The dominant categories for each retaining component (i.e., the variables with the highest value of loading) are presented in Table 2.

**Table 2:** The chosen components for SEVI analysis and their explained variance.

Component	Category	% Variance Explained
1	Demographic Socioeconomic Status	16.15
2	Industrial Economy	14.27
3	Race and Ethnicity	12.13
4	Demographic Socioeconomic Status	10.46
5	Housing and Transportation	9.87
6	Demographic Socioeconomic Status	6.52
7	Age	5.32
8	Gender	3.49

**Figure 2:**
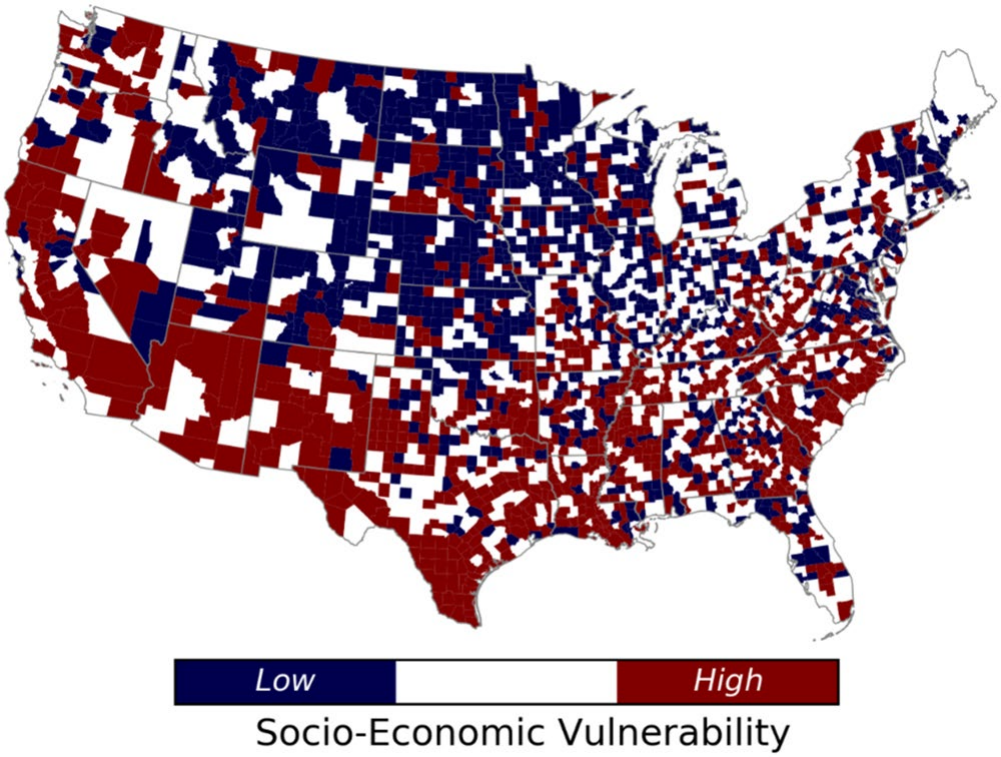
The spatial distribution of Socio-Economic Vulnerability Index (SEVI) at county level across the CONUS.

Results of Table 2 indicate that the demographic socioeconomic status dominates the SEVI with more than 30% of the variance explained. This finding is in agreement with the results from Cutter and Finch^21^, in which the socioeconomic status explained approximately 20% of the variance in five decades at a county level. The calculated SEVI of each county is classified into three classes of high vulnerable, medium vulnerable, and low vulnerable. The low and high vulnerable counties are the ones placed in the 10^th^ and 90^th^ percentile of the calculated index. Figure 2 shows the SEVI at the county level.

As shown in Fig. 2, the most vulnerable counties are concentrated in the southwest and the southern Plains (i.e, Texas and Louisiana) as well as along the U.S.–Mexico border regions of Texas. The least vulnerable counties are located in New England and the upper Great Lakes. Results from the previous assessments^16,21,52^ indicate similar spatial geographical patterns. Cutter and Finch^21^ showed that the vulnerability is increasing in the southwest parts toward the U.S.–Mexico border regions of Texas in the last four decades, which can be due to the clustering of high vulnerable counties in that region, based on the SEVI calculated in this study.

### Spatial distribution of flash flood characteristics

**Figure 3:**
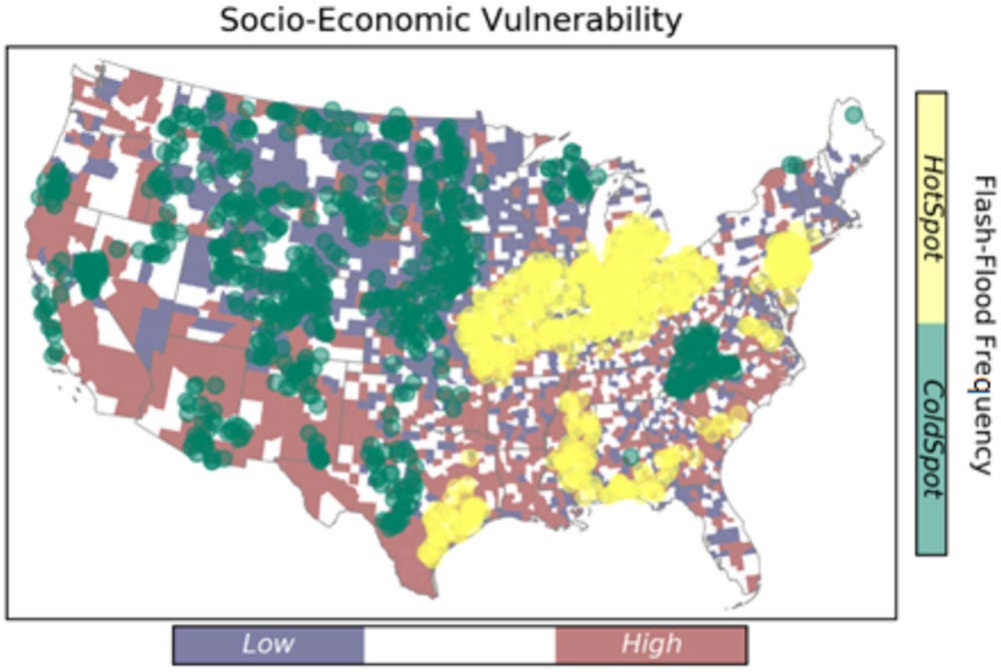
Spatial clustering of flash flood frequency over the CONUS. The underlying map shows the distribution of social vulnerability at county level.

**Figure 4:**
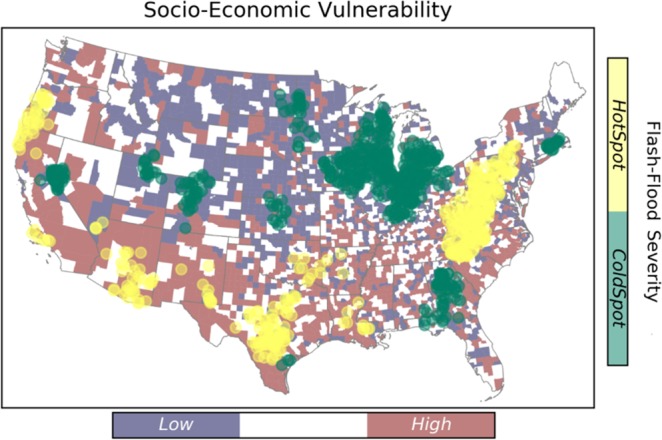
Spatial clustering of flash flood severity over the CONUS.

This study targets four characteristics of flash flood including frequency, magnitude, duration, and severity. Results from the hotspot analysis are summarized in Fig. 3 through Fig. 4. Hotspots represent the spatial clustering of high values for a given characteristic, and the spatial clustering of low values are indicated by the cold spots.

As shown in Fig. 3, the Midwest experiences the largest values of flash flood frequency, whereas the cold spots are located in the Great Plains region. Eastern and southern parts of the U.S. show smaller hotspot clusters, and scattered coldspots are visible in the southwest and pacific regions.

**Figure 5:**
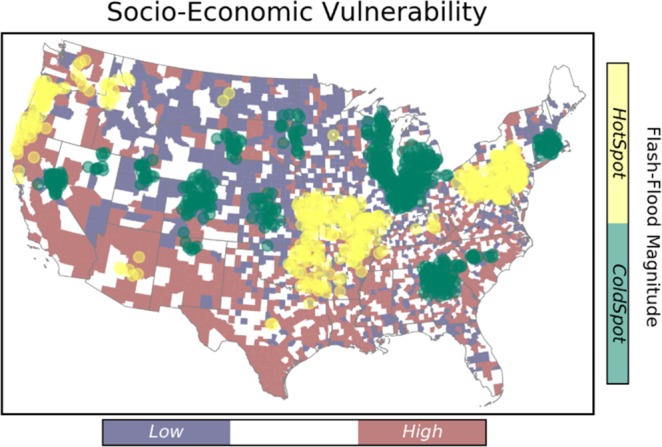
Spatial clustering of flash flood magnitude over the CONUS.

The flash flood magnitude has the smallest clusters comparing to the rest of characteristics (Fig. 5). The hotspot clusters of flash flood magnitude are located in the Missouri Valley, East coast, and Oregon. The largest coldspot of flash flood magnitude is located in the Upper North region. This indicates that the magnitude of flash flood varies substantially throughout the CONUS.

**Figure 6:**
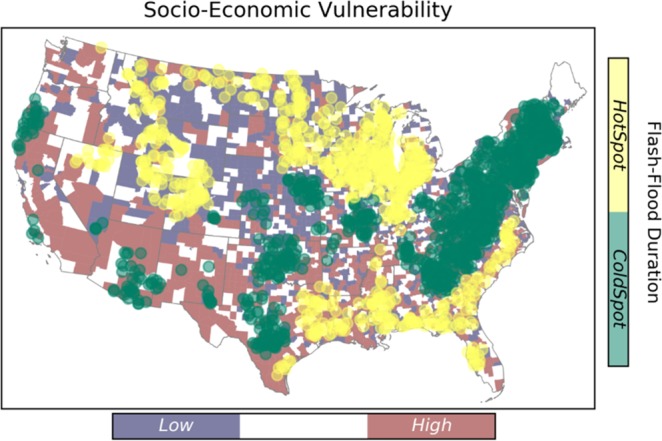
Spatial clustering of flash flood duration over the CONUS.

Figure 6 represents the results for flash flood duration. A considerably large coldspot cluster is located in the Northeastern and part of Southeastern regions. The hotspot clusters are spread out in the lower part of Southeast, Midwest, and Great Plains. Disperse clusters of short flash flood duration are located in the Central U.S., Southwest, and pacific regions.

Similar to the flash flood magnitude, flash flood severity has small number of clusters, as it depends on the magnitude (Fig. 4). The hotspot clusters of flash flood severity are located in the Appalachians, Texas, Arizona, and West coast. On the other hand, the Missouri Valley is where the coldspot of flash flood severity is located. This spatial pattern is observed in a recent study by Saharia *et al*.^64^, and they investigated the flash flood severity over the United States by introducing a new index called the flashiness. Flashiness was defined as the difference between the peak discharge and action stage discharge normalized by the flooding rise time and basin area.

**Figure 7:**
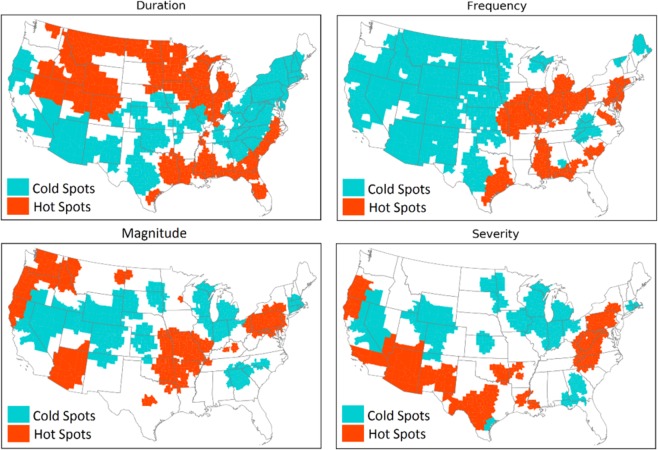
Flash flood hazard characteristics converted from gauge station to the county-scale.

One of the porpuses of this study is to invesigate the coincide of flash flood chararacteristics and socio-economic vulnerability over the CONUS. Therefore, the flash flood characteristis (i.e., frequency, duration, magnitude, and severity) should be converted to county-scale from the station data, which was carried out by employing the overlay tools in ArcMap. Figure 7 represents the flash flood hazard characteristics on the county level. The orange counties are the ones where the stations show hotspots for that specific flash flood characteristic and the blue color indicates the coldspots.

The counties that are located in the Pacific Northwest region indicate hotspots for the flash flood magnitude and severity, whereas it is not the case for the duration and frequency. The majority of counties in the Northern Great Plains are located in the hotspots for flash flood duration and cold spot for frequency. The western and central couties experience less frequent flash flood compared to the Southeast and Northern U.S. The Southwest and Southern Great Plains experienced more severe flash floods with shorter duration.

### Analogy between socio-economic vulnerability and flash flood hazard

**Figure 8:**
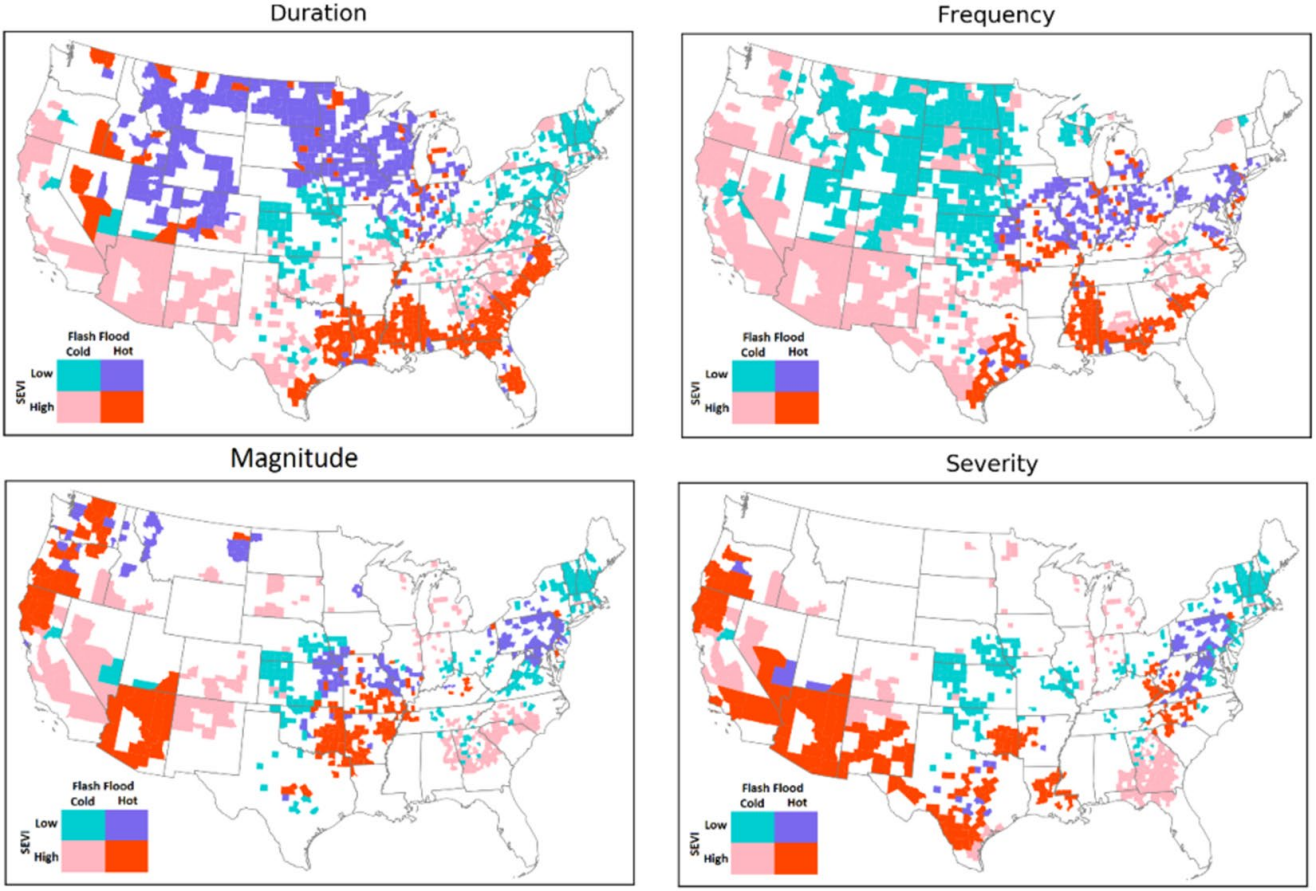
Maps of the counties where flashflood extremes coincide with socio-economic vulnerability extremes.

A two-way cross tabulation is used to map the coincidence of SEVI and flash flood hazard characteristics, and the results are presented in Fig. 8. Comparing the results with respect to flash flood duration and frequency, the high-vulnerable-hotspot counties for frequency are located in the lower Southeast. Whereas, the high-vulnerable-hotspot counties for duration are located in the Southwest and Pacific Northwest.

From Fig. 8, the majority of high-vulnerable-coldspot counties (i.e., the regions associated with high vulnerability and low hazard) are concentrated in the Southwest for flash flood duration, frequency and magnitude, however; they are scattered in regard to severity. The low-vulnerable-coldspot counties are densely located at the Northern Great Plains and Midwest, comparing the frequency with other flash flood characteristics. Focusing on flash flood duration, the low-vulnerable-hotspots are located in the Northern Great Plains. In case of a large flash flood event with vast spatial scale, the hotspot-high vulnerable regions such at Southwest (shown in orange) will probably be affected the most, and since they indicate cluster of areas that will probably be highly impacted, they are unlikely to receive immediate aid from adjacent counties either.

Overall, the majority of areas located in hot spots of rapid floods exhibit low SEVI. However, hot spots and high SEVI do intersect and affect a considerable number of counties in the United States. For instance, counties that are located in the southwest regions are prone to severe floods and they have poor socio-economic status, especially in Nevada. Whereas, the southeastern counties with high SEVI are facing more frequent flooding. The high risk counties in the south-east region indicate an overlapping situation of the hot-spots of flooding duration and frequency. In summary, the counties that are located in these regions can benefit from additional flood management assistance, which are mostly located in the southern United States.

## Concluding Remarks

This study presented a thorough assessment of socio-economic vulnerability and its interaction with four main flash flood characteristics over the CONUS. The evaluation of socio-economic vulnerability usually consists of the construction of indices representing the inherent characteristics or qualities of social systems that are susceptible to a potential impact^12,21,22^. For instance, by understanding the vulnerability of a region to flash flooding, it is possible to assign various evacuation policies for different regions accordingly. A region (i.e. county) with high levels of vulnerability should be asked to evacuate at lower flash flood hazards, compared to the regions that have low vulnerability. In addition, special programs can be initiated to assist the regions that are associated with high level of hazard (frequent and intense flash floods) as well as high vulnerability.

In this study, vulnerability was assessed at the county level by employing 36 social and economic variables. Probabilistic Principal Component Analysis (PPCA) was utilized to quantify socio-economic vulnerability index (SEVI). The flash flood characteristics including frequency, magnitude, duration, and severity were considered for evaluating flash flood hazard. The spatial distribution of flash floods was assessed by using hotspot analysis, and the intersection of SEVI and flash flood characteristics were mapped using the cross-tabulation. The southwest region shows severe flash flooding with high magnitude, whereas the Northern Great Plains indicate lower duration and frequency. Critical counties (high-vulnerable-hotspots) are mostly located in the southern parts of the U.S., whereas the majority of counties in the Northern Great Plains are in the non-critical status.

This study aimed to generate a comprehensive SEVI; however, it still has some limitations. Although social vulnerability indices can efficiently describe broad-scale vulnerability, they fall short on integrating localized information related to exposure, sensitivity, and adaptive capacity that is often better collected using qualitative methods such as empirical methods and surveying^52,65^. Moreover, the calculated SEVI is based on only one point in time (the recent past), and the spatial patterns of socioeconomic vulnerability that were identified in this study represent the recent past. In addition, it would be beneficial to look at the SEVI as the simple weighted sum of the physical variables and compare the outcome with the current SEVI. The socio-economic status and flash flood potential inevitably change over time, due to climate change, migration, and social development. Future studies may consider updating these maps or extending them to longer time frames, especially if/when new extreme events occur. It can also be beneficial to validate the calculated socio-economic vulnerability by comparing the post-hazard outcomes such as economic damages and fatalities with the pre-hazard vulnerability in a spatial context.

Lastly, it is essential to quantify the uncertainty associated with the SEVI, in the future studies. The uncertainty in SEVI can raise from multiple sources including the data used, the chosen variables, period of interest, the methodology, and the weighting technique to integrate the variables and calculate the index. An accurate and reliable cascade of uncertainty for SEVI requires that many permutations of each source of uncertainty be investigated in order to reliably capture the range of uncertainty, and then the fraction of total variance caused by each factor can be determined.
